# Computationally Efficient Assembly of Pseudomonas aeruginosa Gene Expression Compendia

**DOI:** 10.1128/msystems.00341-22

**Published:** 2022-12-21

**Authors:** Georgia Doing, Alexandra J. Lee, Samuel L. Neff, Taylor Reiter, Jacob D. Holt, Bruce A. Stanton, Casey S. Greene, Deborah A. Hogan

**Affiliations:** a Department of Microbiology and Immunology, Geisel School of Medicine at Dartmouth, Hanover, New Hampshire, USA; b Genomics and Computational Biology Graduate Program, University of Pennsylvania, Philadelphia, Pennsylvania, USA; c Department of Biochemistry and Molecular Genetics, University of Colorado School of Medicine, Denver, Colorado, USA; d Department of Systems Pharmacology and Translational Therapeutics, Perelman School of Medicine, University of Pennsylvania, Philadelphia, Pennsylvania, USA; University of California—San Diego

**Keywords:** *Pseudomonas aeruginosa*, RNA-seq, compendium, gene expression, strains, transcriptome

## Abstract

Thousands of Pseudomonas aeruginosa RNA sequencing (RNA-seq) gene expression profiles are publicly available via the National Center for Biotechnology Information (NCBI) Sequence Read Archive (SRA). In this work, the transcriptional profiles from hundreds of studies performed by over 75 research groups were reanalyzed in aggregate to create a powerful tool for hypothesis generation and testing. Raw sequence data were uniformly processed using the Salmon pseudoaligner, and this read mapping method was validated by comparison to a direct alignment method. We developed filtering criteria to exclude samples with aberrant levels of housekeeping gene expression or an unexpected number of genes with no reported values and normalized the filtered compendia using the ratio-of-medians method. The filtering and normalization steps greatly improved gene expression correlations for genes within the same operon or regulon across the 2,333 samples. Since the RNA-seq data were generated using diverse strains, we report the effects of mapping samples to noncognate reference genomes by separately analyzing all samples mapped to cDNA reference genomes for strains PAO1 and PA14, two divergent strains that were used to generate most of the samples. Finally, we developed an algorithm to incorporate new data as they are deposited into the SRA. Our processing and quality control methods provide a scalable framework for taking advantage of the troves of biological information hibernating in the depths of microbial gene expression data and yield useful tools for P. aeruginosa RNA-seq data to be leveraged for diverse research goals.

**IMPORTANCE** Pseudomonas aeruginosa is a causative agent of a wide range of infections, including chronic infections associated with cystic fibrosis. These P. aeruginosa infections are difficult to treat and often have negative outcomes. To aid in the study of this problematic pathogen, we mapped, filtered for quality, and normalized thousands of P. aeruginosa RNA-seq gene expression profiles that were publicly available via the National Center for Biotechnology Information (NCBI) Sequence Read Archive (SRA). The resulting compendia facilitate analyses across experiments, strains, and conditions. Ultimately, the workflow that we present could be applied to analyses of other microbial species.

## INTRODUCTION

The opportunistic pathogen Pseudomonas aeruginosa causes infections in many body sites and is commonly found in chronic lung infections of people with cystic fibrosis (1), where it is difficult to eradicate, and the factors that lead to persistence are not fully understood. P. aeruginosa is also found in soil (2) and freshwater (3, 4), and it is cultured for biotechnology applications (5, 6). The metabolic versatility of P. aeruginosa is partly attributable to adaptive behavioral changes driven by gene expression. Given the unusually high numbers of transcription factors, sigma factors, and two-component systems in the P. aeruginosa genome (7–9), transcriptional profiling across conditions and mutant genotypes has been a fruitful approach to better understand P. aeruginosa physiology. Many P. aeruginosa studies use the laboratory strains PAO1 and PA14 as models for the study of transcriptional regulation, and many studies have also examined gene expression in clinical isolates. The breadth of P. aeruginosa research is reflected in the abundance of transcriptional data sets in public databases, including those hosted by the National Center for Biotechnology Information (NCBI), such as the Sequence Read Archive (SRA) and the Gene Expression Omnibus (GEO), and those hosted by the European Molecular Biology Laboratory European Bioinformatics Institute (EMBL-EBI), such as the European Nucleotide Archive (ENA). To date, over 4,000 P. aeruginosa expression profiles from microarray and RNA sequencing (RNA-seq) technologies are publicly available.

The P. aeruginosa community has long supported the development and widespread use of databases, information hubs, and analysis tools such as the Pseudomonas Genome Database (10), BACTOME (11), the International Pseudomonas Consortium Database (12), the Pseudomonas aeruginosa Metabolome Database (13), the Pseudomonas aeruginosa transcriptome viewer (14), and the shiny applications with the algorithmically annotated data sets GAPE (15) and CF-Seq (16). Tools have also been developed that utilize public data from many experiments in concert, such as the ADAGE Web server, which enables the exploration of P. aeruginosa gene expression microarray data after processing by a machine learning algorithm (17).

**FIG 1:**
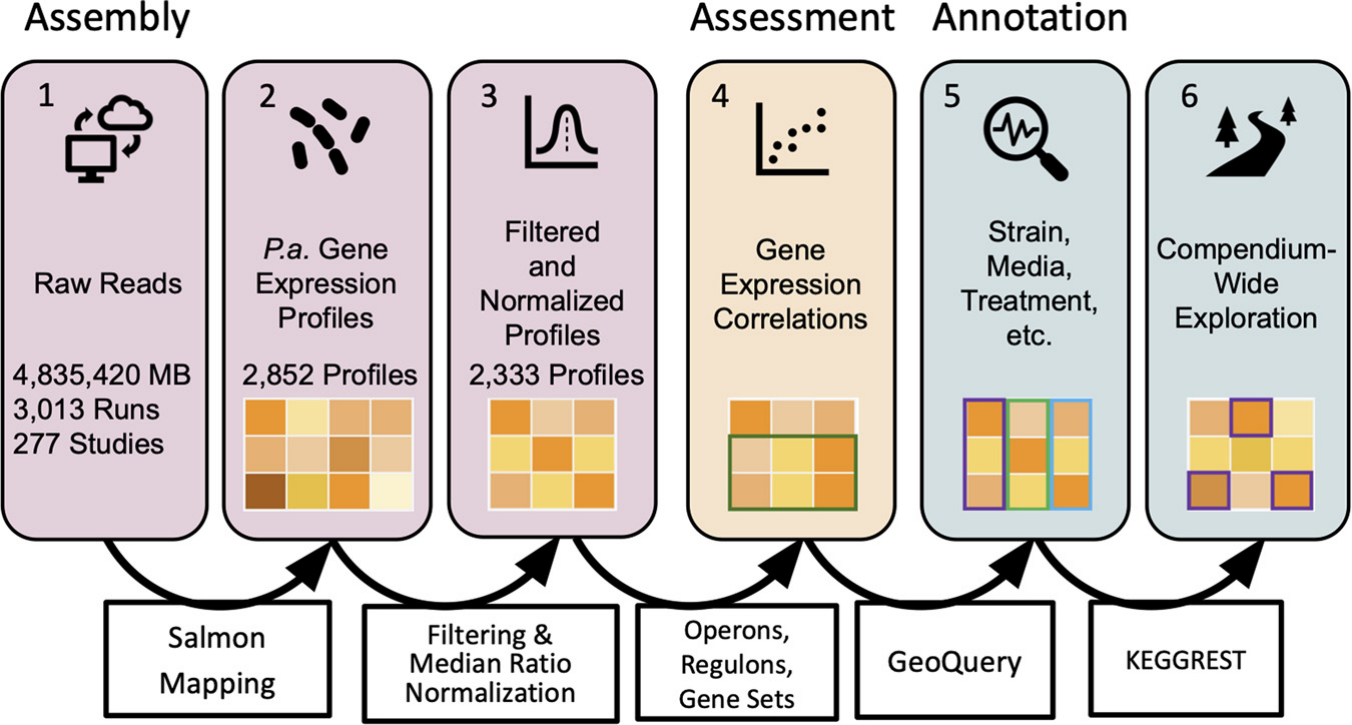
Overview of the steps involved in compendium construction. The steps (numbered boxes) and corresponding methods (boxes with arrows) for assembly (steps 1 to 3), assessment (step 4), and annotation (steps 5 and 6) of a compendium of public RNA-seq gene expression profiles (represented by a matrix of orange squares) for P. aeruginosa are shown. (Step 1) Raw reads from the Sequence Read Archive (SRA) totaled over 4 million MB. “Runs” refers to fastq files, and “Studies” refers to the sets of runs deposited together. (Step 2) P. aeruginosa (*P.a.*) gene expression profiles refer to the results of mapping reads from a sample (referred to in the SRA as “experiment”) to a reference genome and can be read as counts or transcripts per million. (Step 3) Profiles were filtered to remove those that did not meet expression profile criteria and then median ratio normalized. (Step 4) Sets of coregulated genes were used to benchmark target patterns. (Step 5) Samples in the compendium were annotated for strain, media, genetic modifications, treatments, and other fields of interest. (Step 6) Pathway and function data facilitated compendium-wide explorations.

To support the exploration of public RNA-seq data and to further the development of resources that leverage these data, we present a computationally efficient method to reprocess RNA-seq data sets (see Fig. 1 for an overview). After validating a method for high-throughput read mapping of P. aeruginosa data, we collected publicly available P. aeruginosa RNA-seq data, generated gene expression profiles, filtered samples that did not meet quality control metrics, and normalized the data (Fig. 1, steps 1 to 3). We assessed this approach by examining correlations between coregulated genes (Fig. 1, step 4). Finally, we summarized metadata annotations to provide users with information on the composition of the compendium (Fig. 1, steps 5 and 6). Thus, we present a method to build uniform compendia from public data and demonstrate its success using P. aeruginosa gene expression profiles. This method and the resultant compendia are utilized in a companion paper wherein Lee and coauthors use PAO1- and PA14-specific compendia to identify strain-stable gene expression patterns and interrogate transcriptional relationships among and between core and accessory genes (18).

## RESULTS

### Construction of P. aeruginosa gene expression compendia.

The NCBI SRA database was queried for RNA samples of the organism Pseudomonas aeruginosa (query “Pseudomonas aeruginosa” [Organism] AND “biomol rna” [Properties]). This resulted in 3,013 NCBI “run” accession numbers, which were from 2,867 samples (indexed by SRA “experiment” accession numbers). For these samples, each run was downloaded as a fastq file using the SRA toolkit and mapped to both the PAO1 and PA14 transcriptomes (cDNA) using Salmon. All samples were sequenced with Illumina technologies, with read lengths ranging from 50 to 150 bp. After read mapping, read counts (NumReads) and transcripts per million (TPM) were combined into separate raw compendia, one of each from reads that mapped to strain PAO1 and one of each from reads that mapped to strain PA14. Thus, these results do not contain data for genes that are not in either PAO1 or PA14. Each compendium contained 2,852 profiles derived from successfully downloaded and mapped samples; 15 samples were not successfully retrieved and were not included in subsequent analyses.

### Assessment of Salmon mapping relative to field-standard alignment for the analysis of P. aeruginosa transcriptional profiles.

Pseudoalignment algorithms such as Salmon (19) estimate transcriptional profiles from high-throughput sequencing reads in a fraction of the time required for traditional alignment algorithms, thereby making the reprocessing of thousands of RNA-seq data sets practical (19, 20). Pseudoalignment has been thoroughly validated and widely used on eukaryotic RNA-seq data (21, 22). However, pseudoalignment algorithms have been less widely used in microbial research, perhaps due to the small size of microbial genomes and, thus, manageable processing times for the average experiment with 4 to 24 samples. While we had no reason to suspect that Salmon pseudoalignment would not be effective for microbial RNA-seq, since it has not been widely used in the microbiology community, we evaluated pseudoalignment by Salmon mapping for the analysis of P. aeruginosa gene expression data using a small number of samples. This is an important first step since the speed and efficiency of pseudoalignment are critical for the processing of thousands of samples for the creation of a filtered, normalized compendium of publicly available RNA-seq data.

To assess the results of RNA-seq data mapping using the Salmon pseudoaligner, we compared the Salmon transcript abundance estimates to the results from the field-standard aligner CLC Genomics Workbench version 12.0 (CLC). We used CLC as a “gold standard” because it uses a traditional full-alignment algorithm against the full genome sequence rather than k-mer hash mapping against a cDNA sequence reference, as Salmon does. For this, we used original samples that we collected under conditions designed to elicit well-characterized transcriptional differences: P. aeruginosa wild-type (WT) strain PA14 and a *pstB*::Tn*M* mutant derivative grown as colony biofilms on minimal medium. We chose this comparison because *pstB* mutants have a constitutively active transcriptional response that promotes phosphate scavenging (23), which is driven by the transcription factor PhoB (24). A *pstB* mutant gives a clear signal in differential expression (DE) analyses and can be interpreted in the context of previous RNA-seq experiments by the Häussler group characterizing the low-phosphate response (24, 25). We processed each of the wild-type and *pstB* mutant samples using both Salmon and CLC.

An important parameter for Salmon pseudoalignment is the “library type,” which is determined by whether the reads were obtained via paired-end or single-end (unpaired) sequencing. Publicly available P. aeruginosa RNA-seq data consist of a mix of paired and unpaired reads. The libraries from the wild-type and *pstB*::Tn*M* samples contained paired-end reads, so we compared the results of mapping with the library-type flag set to “paired” and “unpaired.” When the data were mapped specifying the library type as paired, Salmon had lower estimates than CLC for the expression of many genes, especially those that were lowly expressed. This difference was not observed when the library type was specified as unpaired (see Fig. S1 in the supplemental material). Linear models for the comparison of CLC- and Salmon-generated TPM in the paired mode (average adjusted *R*^2^ value across samples of 0.66) showed a worse fit than those in the unpaired mode (average adjusted *R*^2^ value of 0.76). We suspect that differences in TPM values between Salmon and CLC were due to the presence of polycistronic transcripts, which are very common in bacterial transcriptomes but violate assumptions made by Salmon for paired-end libraries when a cDNA-based reference transcriptome is used. Pseudoalignment algorithms precompute transcriptome indices for k-mer mapping of reads. When genes are in operons, the assumption that forward and reverse reads (each 50 to 150 bp long) would map to the same transcriptome index (same cDNA sequence) does not necessarily hold since each gene, not mRNA, corresponds to its own index. If a pair of reads spans the junction of two genes, the forward and reverse reads could map to different transcriptome indices. CLC does not encounter this challenge since it aligns to a full-genome reference with coding sequences annotated. Thus, when a read spans two genes in an operon, it can be assigned to the gene with the longer alignment segment rather than being discarded for ambiguity (CLC Genomics Workbench Manual). To determine if the improved concordance between Salmon and CLC with the unpaired mode was due to the improved performance on polycistrons, we analyzed the correlations between mapping methods for monocistronic genes and polycistronic operons separately. We found that both mono- and polycistronic operons had better fits between CLC- and Salmon-generated data when processed in the unpaired mode, but polycistrons benefited more from the use of the unpaired mode (an increase of the adjusted *R*^2^ value from 0.83 in the paired mode to 0.91 in the unpaired mode) than did monocistrons (an increase of the adjusted *R*^2^ value from 0.83 in the paired mode to 0.89 in the unpaired mode). Overall, treating the data as unpaired improved the concordance for all genes regardless of the operon size. While treating all data as unpaired does not reflect the actual nature of the data, these empirical results show that it provides a work-around for the shortcomings in the performance that Salmon achieved compared to CLC, and therefore, we maintained the parameter of unpaired reads for all data sets regardless of the library type.

10.1128/msystems.00341-22.1 FIG S1 Salmon parameter choice of unmapped reads. A comparison of transcripts per million (TPM) for a single P. aeruginosa strain PA14 RNA-seq sample (WT) (rep 1) aligned using CLC to TPM determined by Salmon in mapping mode with the library type specified as either paired end (paired) (orange) or unpaired (purple) was performed. When the library type was specified as “paired,” Salmon underestimated some transcripts compared to CLC, reflected in the lower adjusted *R*^2^ value determined by the linear model of log_10_ TPM data. Download FIG S1, TIF file, 2.6 MB. Copyright © 2022 Doing et al. 2022 Doing et al. https://creativecommons.org/licenses/by/4.0/ This content is distributed under the terms of the Creative Commons Attribution 4.0 International license.

### Salmon mapping and CLC alignment produce similar differential expression data.

**FIG 2:**
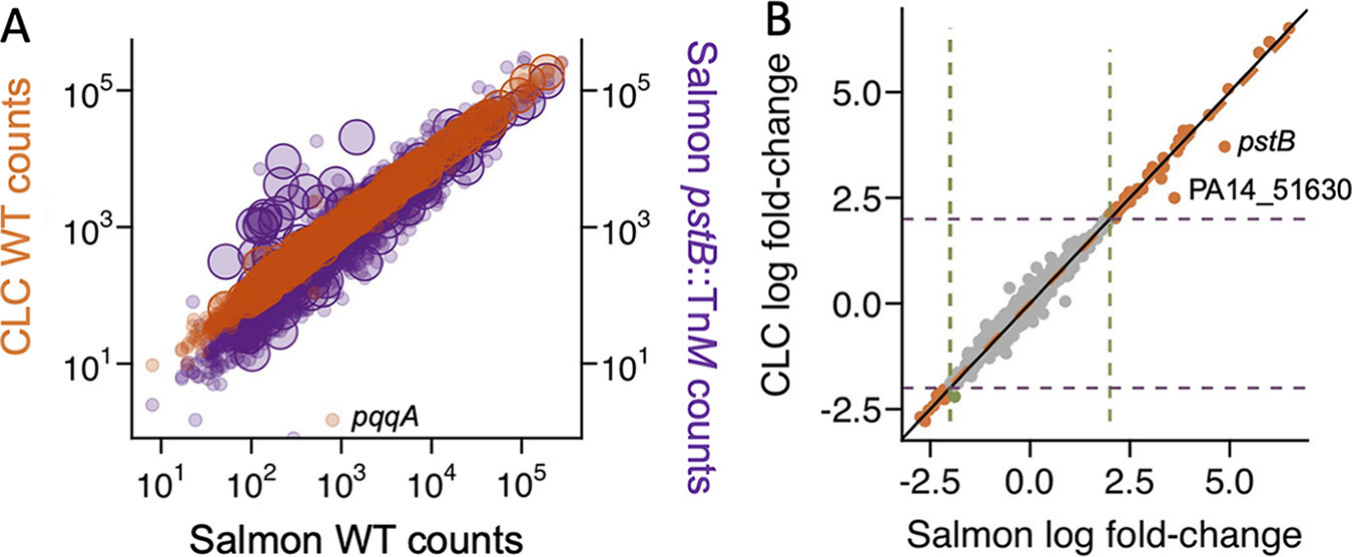
Validation of the Salmon pseudoalignment method for the analysis of P. aeruginosa RNA-seq data. (A) Log_10_ raw counts [log(counts)] for wild-type P. aeruginosa grown in MOPS minimal medium with 0.7 mM phosphate (*n* = 2) as determined by Salmon were highly similar to those determined by CLC (left y-axis, orange). The variation in the data across the two methods (adjusted *R*^2^ = 0.99) was less than the fold-change between the wild type and the *pstB*::Tn*M* mutant grown under the same conditions (*n* = 2) when all samples were analyzed by Salmon (right y-axis, purple) (adjusted *R*^2^ = 0.72). PhoB-regulated genes, the levels of which were expected to be higher in the *pstB*::Tn*M* mutant, are indicated with larger circles. (B) Log_2_ fold change values between the wild type and the *pstB*::Tn*M* mutant were determined by both Salmon and CLC. Points with an absolute log_2_ fold change value of >2 by both methods are indicated in orange. Three genes (PA14_07380, PA14_19680, and PA14_62690) had absolute log_2_ fold change values of >2 by CLC but not Salmon (overlapping green points). In a linear model using log_2_ fold change values from all genes, the adjusted *R*^2^ value was 0.98.

To further evaluate Salmon quantification for the analysis of P. aeruginosa RNA-seq data, we performed DE analyses comparing the expression profiles of P. aeruginosa wild-type strain PA14 and a *pstB*::Tn*M* mutant and looked for the expected differences in the PhoB-controlled low-phosphate response (23). Alignment by CLC and mapping by Salmon of the P. aeruginosa WT and *pstB*::Tn*M* data sets to the strain PA14 cDNA reference genome generated read count values per gene that were highly similar for the two mapping methods (Fig. 2A, orange symbols, and Data Set S1). The differences between the algorithms used in these two methods make it impressive that we see high concordance in the data analyzed by these two approaches. The correlation for a sample analyzed by the two different alignment methods was very high (adjusted *R*^2^ = 0.99). For comparison, the correlation between the WT and *pstB*::Tn*M* samples analyzed by Salmon, which reflects the differential expression signal, had an adjusted *R*^2^ value of 0.72 (Fig. 2A, purple symbols). Only one gene, *pqqA*, which is a short, 72-nucleotide (nt)-long gene, appeared to have higher counts estimated by Salmon than by CLC (Fig. 2A), which may be related to the fact that there are multiple segments of the *pqqA* gene that have 90 to 100% identity over >20 nt with other P. aeruginosa genes as determined by BLASTN.

10.1128/msystems.00341-22.4 DATA SET S1 WT versus *pstB* differential expression analyses. (Sheet 1) Comparison of the CLC full alignment and Salmon mapping (pseudoalignment) preprocessing effects on downstream differential expression analyses comparing P. aeruginosa WT PA14 grown with sufficient phosphate to suppress the low-phosphate response and the mutant derivative with a disrupted repressor, *pstB*::Tn*M*, thus having a constitutive low-phosphate response under these conditions. (Columns A to F) Annotations of PA14 locus tags, PAO1 homologs (note that PA14 loci can map to multiple PAO1 homologs, leading to duplicate PAO1 loci such as PA0263 and PA2170), gene names, and whether each gene is regulated by PhoB in response to low phosphate. (Columns G to U) EdgeR differential expression analysis results for the WT versus the *pstB*::Tn*M* mutant were aligned to the PA14 reference genome in CLC and Salmon and aligned to the PAO1 reference genome in Salmon. RNA was extracted from colony biofilms grown on a solution containing MOPS minimal medium, 0.7 mM P_i_, and 0.2% glucose. (Columns V to AG) Raw count values for WT and *pstB*::Tn*M* samples used in the differential expression analyses. (Columns AH to AS) TPM values for WT and *pstB*::Tn*M* samples used in the differential expression analyses. (Sheet 2) Genes with identical paralogs in either strain PAO1 or strain PA14. Download Data Set S1, XLSX file, 2.1 MB. Copyright © 2022 Doing et al. 2022 Doing et al. https://creativecommons.org/licenses/by/4.0/ This content is distributed under the terms of the Creative Commons Attribution 4.0 International license.

As expected, genes regulated by PhoB (24) showed higher expression levels in the *pstB*::Tn*M* mutant than in the wild type (Fig. 2A, large circles), and they included genes that encode a sensor histidine kinase (*phoR*), an alkaline phosphatase (*phoA*), a periplasmic phosphate-sensing appendage (*pstS*), machinery for the import of both phosphate and phosphonate (*phnC*), secreted phosphate scavengers such as phospholipase C (*plcN*) and an extracellular DNase (*eddA*), as well as an extracytoplasmic sigma factor (ECF) known to interact with PhoB in the RNA polymerase holoenzyme (*vreI*) (Data Set S1). Differential expression analysis using EdgeR (26) produced similar per-gene fold change values with data from CLC and Salmon (adjusted *R*^2^ = 0.98) (Fig. 2B and Data Set S1). We also compared the false discovery rate (FDR)-corrected *P* values for the differential expression determined with data processed using either Salmon or CLC. FDR values produced by the differential expression analyses of data processed by either Salmon or CLC correlated with an adjusted *R*^2^ value of 0.81, and differences were largely due to genes that were not significantly differentially expressed (FDR > 0.05). CLC identified 70 genes with significance scores below the common threshold of an FDR of <0.05 that Salmon did not (Data Set S1), which suggests that some exploratory analyses may consider values above this common significance threshold, while follow-up experiments may benefit from larger sample sizes, depending on the method of read mapping used.

Two genes met the standard fold change cutoff by both methods but were underestimated by CLC compared to Salmon (Fig. 2B): *pstB* (PA14_70810 [PA5366]), which contained a transposon insertion in the *pstB*::Tn*M* samples, and PA14_51630 (PA0978), which encodes a transposon-associated integrase with high-identity sequences elsewhere in the genome. Prompted by the differences in genes with high-identity sequences, we analyzed the genomes for identical paralogs within both the PAO1 and PA14 genomes. We found 34 loci for which there were identical or nearly identical paralogs in both strains PAO1 and PA14 (e.g., *phzC1* through *G1* and *phzC2* through *G2*, *tufA* and *tufB*, transposase- and integrase-encoding genes, and *vrgG* and *hcp*). We also found three loci that had identical paralogs in one strain but not the other. These genes are listed in Data Set S1 and were removed from or flagged in analyses, as indicated. Only three genes (PA14_07380, PA14_19680, and PA14_62690) had log_2_ fold change values that were lower than the lower boundary of the frequently used, but arbitrary, absolute log_2_ fold change value cutoff for differentially expressed genes (DEGs) of 2 by CLC but not Salmon (Fig. 2B, overlapping green points), but they were very close to the threshold. Overall, these data suggest very high concordance between differential gene expression analyses performed on data mapped by CLC and Salmon.

### Feasibility of using a single reference genome for expression analyses across strains.

Genome analyses of thousands of environmental and clinical isolates of P. aeruginosa have revealed a population structure that includes a number of distinct clades (10). Numerous strains from different P. aeruginosa clades have been analyzed by RNA-seq. We sought to analyze data for these diverse strains using a common reference genome for simplicity of data processing and to obtain data with common gene nomenclature. P. aeruginosa genome variation, both within and between clades, includes differences in accessory gene contents (27). For example, when the genomes of two strains from different clades, PAO1 and PA14, are compared, 58 PA14 regions (containing 478 genes) were absent in PAO1, and 54 PAO1 regions (containing 234 genes) were absent in PA14 (28). Some “strain-specific” genes encode protein homologs known to vary markedly across strains, such as extracellular components of type IV pili and the flagellum (28) and pyoverdine biosynthesis enzymes. Other strain-specific genes encode proteins that participate in processes carried out by enzymes that differ markedly across strains, such as restriction-modification systems, pyocins, and serogroup-specific lipopolysaccharide biosynthesis loci (29). An analysis of five strains by Mathee et al., which included strains PAO1 and PA14, found that 90% of P. aeruginosa genes (>5,100 genes) have more than 75% identity between strains, the majority of which (>4,500 genes) have at least 99% identity (30). In contrast to the coding sequences, there are lower levels of sequence identity in intergenic regions, and these regions were excluded from our analyses using cDNA reference genomes. In this study, we focused on creating RNA-seq compendia of 2,333 samples all mapped to both the PAO1 and PA14 cDNA reference genomes and validating these compendia using expression patterns from gene sets comprised of core genes present in both strains. We analyze the relationships between the expression of core genes and accessory genes in both strains PAO1 and PA14 in the companion article by Lee et al. (18).

**FIG 3:**
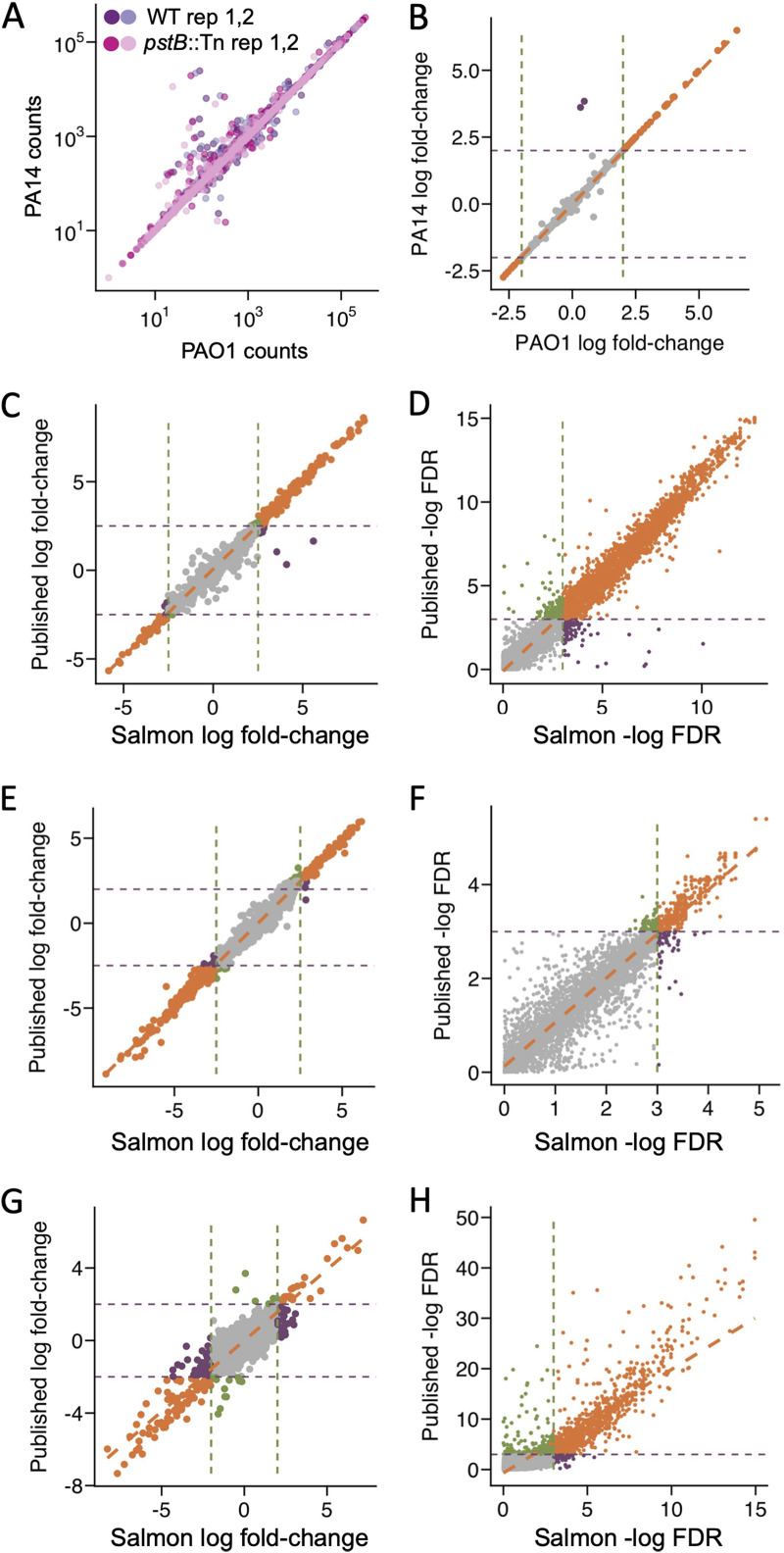
Analyses of the effects of the reference genome on expression analysis. (A) Mapping of P. aeruginosa WT strain PA14 and *pstB*::Tn*M* reads using PAO1- or PA14-based transcriptional indices showed that some genes had more reads (counts) for the PA14 genome, while other genes had more reads for the PAO1 genome. (B) Most high-magnitude values, with log_2_ fold changes of >2, derived from PAO1-mapped data were highly similar to those derived from PA14-mapped data (orange), and two genes had high-magnitude fold change values by PA14-mapped but not by PAO1-aligned data (purple). (C) For samples with GEO series accession number GSE55197 using strain PA14 grown in phosphate-replete and low-phosphate media, most log_2_ fold change values from data aligned to the PA14 cDNA reference genome by Salmon were highly similar to those from published count tables, including most genes with values above the common cutoff of 2 in either analysis (orange). The genes with high log_2_ fold change values by the Salmon method but not in published data were among those with high-identity paralogs, as described above (purple). (D) For the experiment shown in panel C for differential expression analyses of Salmon-mapped data and data from public count tables, the −log_10_ FDR values for the duplicate samples were similar. (E) Samples with GEO series accession number GSE142448, which describes the P. aeruginosa response to manuka honey, analyzed using Salmon with the strain PA14 cDNA reference showed high congruence in log_2_ fold changes to data in published count tables. (F) For the manuka honey experiment shown in panel E done in triplicate (*n* = 3), the −log_10_ FDR values from differential expression analyses are highly similar, especially at lower values, between Salon-mapped data and data from public count tables. (G) Samples from the experiment with GEO series accession number GSE68534 comparing clinical isolate J215 and its Δ*anr* derivative mapped to PAO1 showed high congruence in fold changes between Salmon-mapped data and published counts. Genes near the arbitrary threshold that met the log_2_ fold change cutoff by the Salmon method but not the published count tables (purple) or the by the published count tables but not the Salmon method (green) are shown. (H) As for PA14-mapped data, −log_10_ FDR values from Salmon-mapped data and data from published count tables were similar, with some skewing at the lowest values.

To experimentally assess the feasibility of mapping P. aeruginosa samples from different strains to a common reference genome, we compared the read counts for each gene after mapping WT PA14 and *pstB*::Tn*M* RNA-seq data against the cDNA reference genomes for both PA14 and PAO1. We found that the Salmon-mapped results for homologous (core) genes were highly similar regardless of whether the PAO1 or PA14 reference genome was used (average adjusted *R*^2^ value of 0.93 [range of 0.91 to 0.94 for log counts across all four samples]) (Fig. 3A), which is consistent with the fact that the average nucleotide identity for homologs across these two strains is 99.1% (28, 31). After excluding genes present in multiple copies in a single genome, for which reads could not be mapped accurately, genes that yielded different counts and TPM depending on which reference genome was used included gene sequences known to vary across strains (e.g., the gene that encodes the type IV pilin, *pilA*, and pyocin-encoding genes) or genes that may have difficult-to-map regions (e.g., PA0690, which encodes a large hemagglutinin with many repeats). As it is more likely that these differences are at least partly driven by technical or sequence-driven differences in the alignment, expression differences for these loci require additional consideration by investigators. An in-depth analysis of differences in gene expression patterns between PAO1- and PA14-specific compendia is pursued in the companion article by Lee and coauthors (18), who identify strain-stable and strain-variable patterns in core and accessory genes in PAO1 and PA14.

In differential expression analyses between the wild-type and *pstB*::Tn*M* strains, using Salmon-mapped data with the PAO1 and PA14 cDNA reference genomes, only two core genes from a single operon (PA14_51620 [PA0978] and PA14_51630 [PA0979]) had high-magnitude log_2_ fold change values when the data were aligned to the PA14 reference cDNA genome (log_2_ fold changes of 3.84 and 3.61, respectively) but not when the data were aligned to that of strain PAO1 (log_2_ fold changes of 0.47 and 0.32, respectively) (Fig. 3B). These loci were among those that had multiple paralogs in one genome but not the other, and thus, they were not considered further (Data Set S1). In the companion article that examines compendia of all public PAO1 and PA14 samples, Lee and coauthors show via principal-component analysis (PCA) that there was not a strong separation of PAO1- and PA14-derived samples (using both core and accessory genes) and that the distributions of sample types were very similar in PCA plots generated for both compendia regardless of whether the PAO1 or PA14 reference genome was used (18). These findings strongly support the notion that gene expression analyses can be performed across strains (at least for PAO1 and PA14) and that transcriptional patterns reflect the expression of highly similar core genes that strains have in common.

Expanding our validation of Salmon to public data sets, we identified published studies that deposited their count tables in GEO and compared the data from their DE analyses to the data obtained using our Salmon-based mapping workflow. The results from the reanalysis of strain PA14 samples grown in phosphate-replete and low-phosphate media from a previous study by the Häussler group examining the transcriptional responses to different conditions (GEO series accession number GSE55197) (25), which used the PA14 cDNA reference genome but a different alignment algorithm, were very strongly congruent with the published results in terms of log_2_ fold changes (*R*^2^ = 0.98) (Fig. 3C) and −log_10_ FDR-corrected *P* values (*R*^2^ = 0.82) (Fig. 3D). Again, the three genes standing out as having high log_2_ fold change values in Salmon-mapped data but not in published count tables (Fig. 3C, purple symbols) were genes present in multiple copies, and thus, they were not considered further (Data Set S1). The results from reprocessing data from another study (GEO series accession number GSE142448), which was reported by Bouzo et al. in a paper examining the effects of manuka honey on the P. aeruginosa transcriptome (32), using our Salmon workflow also closely matched the published data for log_2_ fold changes (*R*^2^ = 0.99) (Fig. 3E) and −log_10_ FDR-corrected *P* values (*R*^2^ = 0.84) (Fig. 3F). Finally, we demonstrated that our Salmon-based workflow obtained results that were similar to published data (GEO series accession number GSE68534) for a clinical isolate (strain J215) and its Δ*anr* derivative (33) upon mapping to the PAO1 reference genome for log_2_ fold changes (*R*^2^ = 0.85) (Fig. 3G) and −log_10_ FDR-corrected *P* values (*R*^2^ = 0.67) (Fig. 3H). Taken together, our Salmon-mapped data produced DE results similar to those derived from published count tables generated using other methods across data produced by our laboratory and others using different strains.

### Heuristic-based filtering of the data to improve cross-experiment comparisons.

To ensure that technical factors such as average read depth or DNA contamination do not unduly influence data interpretations, we implemented compendium-wide filters to ensure that all transcriptional profiles included in the final compendia met a uniform set of standards. Thus, we excluded profiles based on two characteristics: sparsity (the number of genes with zero counts, representing undetected transcripts) and the median expression values of a set of nine housekeeping (HK) genes (*ppiD*, *rpoD*, *proC*, *recA*, *rpsL*, *rho*, *oprL*, *tpiA*, and *nadB*). These criteria were chosen as factors that can dominate gene expression patterns, may be driven by technical factors, and would have an undue influence on the interpretation of the results.

**FIG 4:**
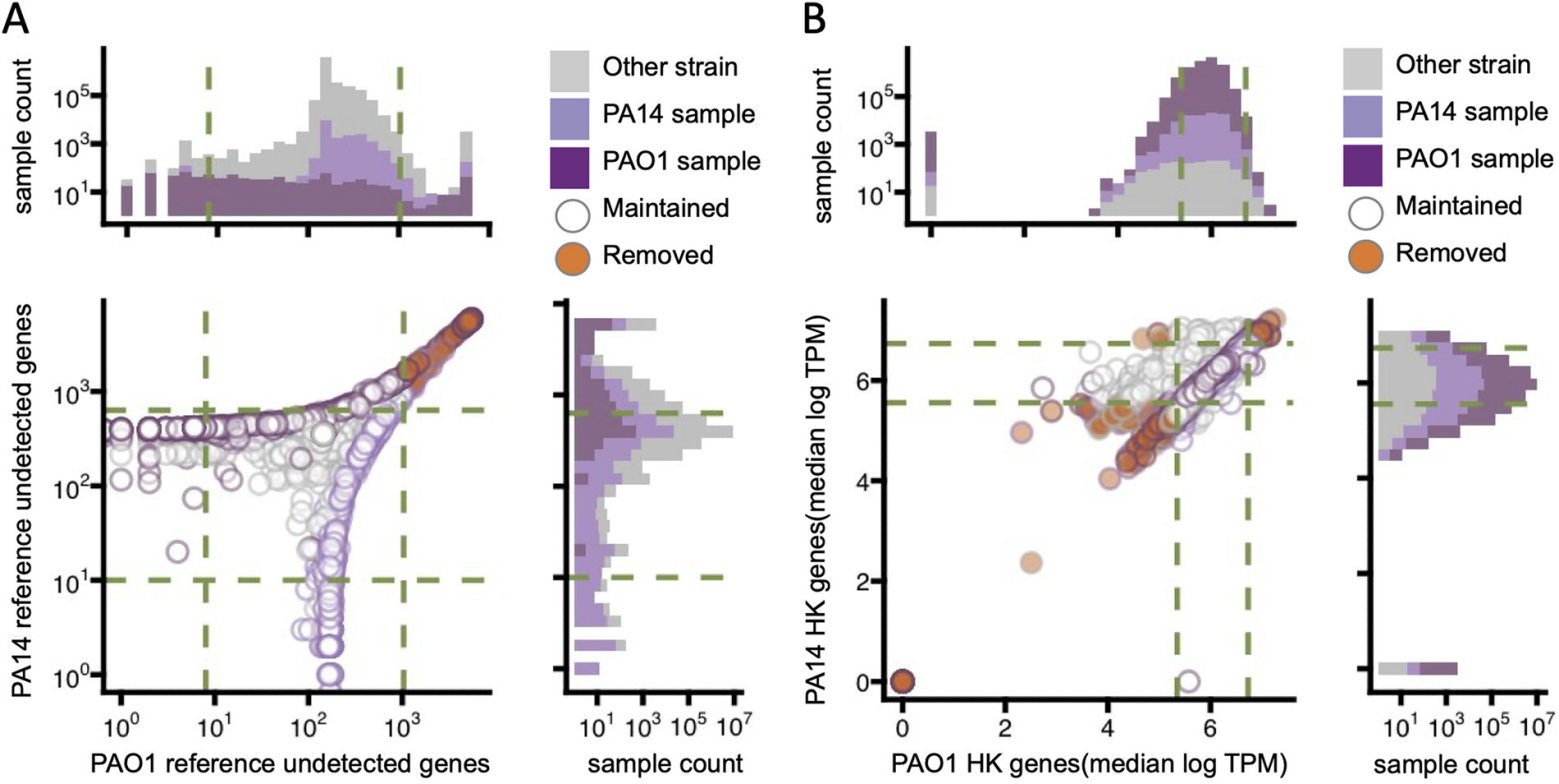
Compendium filtering guided by distributions of sparsity and housekeeping gene expression. (A) Filtering thresholds for sparsity were defined as the 10th and 90th percentiles for PAO1 and PA14, separately determined for samples annotated as belonging to each strain, as indicated by circles showing PAO1 (dark purple), PA14 (light purple), or otherwise annotated or unannotated samples (gray). Samples were excluded (removed) (orange-filled circles) from the compendia if they fell outside the ranges by both PAO1- and PA14-defined criteria; otherwise, they were included (maintained) (unfilled circles). The top and side histograms show profile counts. (B) Filtering thresholds for housekeeping gene expression were defined as the 20th and 98th percentiles using the same samples as the ones used for the sparsity threshold determinations. Strain annotations and inclusion (maintained) or exclusion (removed) are indicated by the circle outlines and fill colors as described above for sparsity filtering. The top and side histograms show profile counts.

The numbers of undetected transcripts per sample were very similar in both the PAO1-mapped and PA14-mapped compendia. For each prefiltered, prenormalized compendium, we generated histograms showing the number of undetected genes per profile, and we analyzed these parameters for the subsets of samples known to be derived from these P. aeruginosa strains based on metadata annotations (Fig. 4A). This analysis did not reveal any obvious trends based on strain in either prefiltered, prenormalized compendium aside from slight skews of PA14 samples having more undetected genes when mapped to the PAO1 reference than PAO1 samples and vice versa when mapped to a PA14 reference, consistent with the known numbers of PAO1- and PA14-specific genes (Fig. 4A, histograms). Above the 90th percentiles, the profiles showed a very strong correlation between the number of undetected genes in the PAO1-mapped compendium and the number of undetected genes in the PA14-mapped compendium, indicating that these values reflect technical features of the sample and are not due to differences in gene content. We thus chose thresholds for sparsity at the 10th and 90th percentiles. The profiles removed based on these criteria are indicated in Fig. 4A by filled-in circles (orange). These thresholds aimed to exclude the population of profiles that were very sparse and produce a filtered compendium with a more normal distribution of sparsity characteristics. After filtering, the profiles had between 8 and 1,037 undetected transcripts in the PAO1-mapped filtered compendium and between 10 and 1,037 undetected transcripts in the PA14-mapped filtered compendium.

The median expression values of the nine HK genes were also determined for every profile in both the PAO1- and PA14-mapped compendia. Again, the ranges of values were similar across the two compendia for PAO1 and PA14 samples. We chose thresholds for median HK gene expression at the 20th and 98th percentiles (Fig. 4B, removed samples marked with orange symbols), and these values were chosen partly to remove profiles with very low expression levels of genes that are generally highly expressed to produce a more uniform final compendium. Additionally, these values were chosen because many of the profiles outside this range were collected for purposes other than transcriptome analyses. For example, samples filtered out based on these criteria included samples under SRA BioProject accession number PRJNA379630 that were generated for ribosomal profiling (34), samples under SRA BioProject accession number PRJNA561330 that were used for RNA immunoprecipitation sequencing (RIP-seq) (35), and samples under SRA BioProject accession number PRJNA439811 that were generated for global small RNA target identification by ligation and sequencing (GRIL-seq) (36) (Data Set S2). All other profiles from samples that were generated using these methodologies were also removed from the filtered compendia. After filtering, profiles in the compendia contained 211 to 840 median HK TPM and 258 to 841 median HK TPM in the PAO1-mapped and PA14-mapped compendia, respectively.

10.1128/msystems.00341-22.5 DATA SET S2 Experiment profile characteristics. Filter criterion (housekeeping gene expression and zero count) values, median accessory gene expression values, and experiment (profile)-wise annotations for the prefiltered compendium are shown. Download Data Set S2, XLSX file, 1.1 MB. Copyright © 2022 Doing et al. 2022 Doing et al. https://creativecommons.org/licenses/by/4.0/ This content is distributed under the terms of the Creative Commons Attribution 4.0 International license.

Of the 2,852 samples processed with Salmon, 2,333 profiles were retained after filtering. There were a few instances of entire experiments being removed, and many profiles that were filtered out were likely outliers within their originally published data sets. We carried on these 2,333 samples, which were mapped to PAO1 reference, through normalization (described below) to ultimately yield the PAO1-mapped compendium. The same set of samples was also mapped to the PA14 reference genome and carried on through normalization (described below) to yield the PA14-mapped compendium. Both the PAO1- and PA14-mapped compendia had 2,333 profiles after filtering.

Sparsity, HK gene expression values, and filtering results for each profile are included in Data Set S2. Because the heuristic-based filtration criteria are not direct measures of the technical quality of a sample or any certain technological bias, consistent with the low numbers of profiles being removed per experiment, filtering cutoffs may be adjusted and optimized for different downstream uses such as those outlined in Discussion.

### Filtering and normalization expose gene expression correlations of coregulated gene sets.

**FIG 5:**
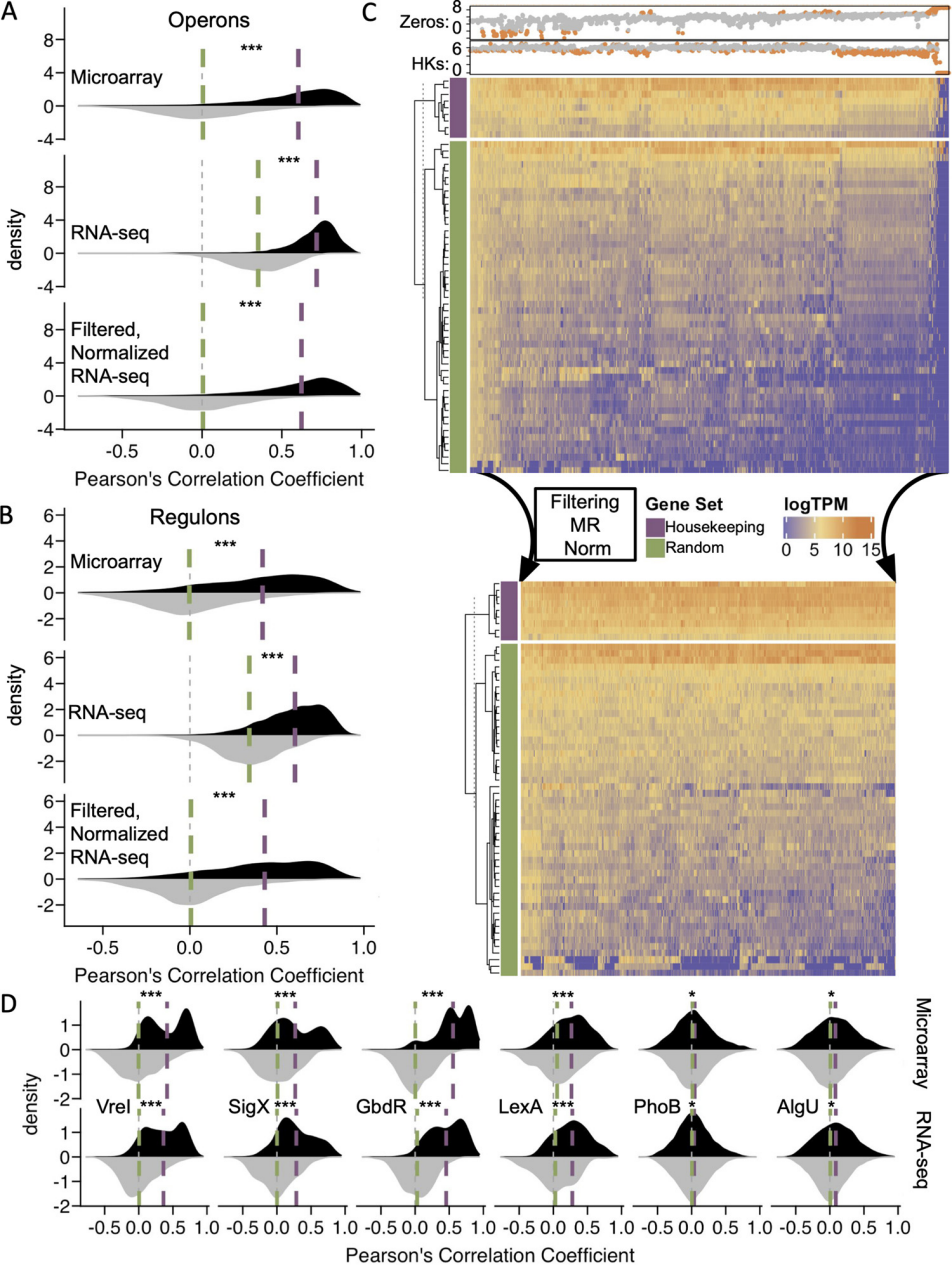
Filtering and normalization remove common patterns of gene expression that manifest as random correlations and maintain gene-gene correlations between coregulated genes in the PAO1-mapped compendium. (A) Similar to a previously published array compendium, profile filtering and median ratio (MR) normalization correct spurious correlations in the expression of random sets of genes (gray distributions), shifting the medians (green lines) to zero and exposing interoperon gene-gene correlations (black distributions) with elevated medians (purple lines). ***, FDR of <0.01 and large effect size (>0.5) (by a paired Wilcoxon test). (B) Interregulon gene-gene correlations show improvements upon filtering (removal of samples outside zero counts [Zeros] and median housekeeping [HK] gene thresholds) and MR normalization similar to those for size-matched random controls. ***, FDR of <0.01 and a large effect size (>0.5) (by a paired Wilcoxon test). (C) After filtration and MR normalization, samples across the compendium have more similar overall distributions of log_10_ TPM for HK (purple) and 50 randomly selected (Random) (green) genes. The top annotations of the unfiltered, unnormalized compendium (top heat map) show log_10_ counts of undetected transcripts per profile (Zeros) and median log_10_ TPM of HK genes colored by whether they were maintained (gray) or removed (orange) in the filtered, normalized compendium (bottom heat map). (D) Within-gene-set gene-gene correlation patterns are similar across the microarray (top) and filtered, normalized RNA-seq compendium (bottom) in a targeted exploratory analysis of transcription factors of interest (black) compared to size-matched sets of random genes (grey). *, FDR of <0.01 and small effect size (<0.25); ***, FDR of <0.01 and large effect size (>0.5) (by a paired Wilcoxon test).

As we had done previously using a compendium of microarray data (37), we analyzed the correlations of gene expression between cooperonic genes (determined as described in Materials and Methods) versus random pairs. We first conducted these analyses using the previously published microarray data compendium (37) in which expression is analyzed by hybridization rather than by sequencing and found higher median cooperonic correlation values (Pearson correlation coefficient of 0.66) than those for random pairs (Pearson correlation coefficient of −0.008) (Fig. 5A), as expected. However, in the prefiltered, prenormalized, PAO1-mapped RNA-seq compendium that we constructed, even random gene pairs were highly correlated (Pearson correlation coefficient of 0.42), although the average correlation for cooperonic genes was still higher (Pearson correlation coefficient of 0.75). The high correlations between randomly chosen genes were similar in both the PAO1-mapped and PA14-mapped compendia, and correlations were evident both before and after the filtering steps described above (Fig. S2A), which suggested a need for normalization.

10.1128/msystems.00341-22.2 FIG S2 Filtering and normalization remove common patterns of gene expression that manifest as random correlations and maintain gene-gene correlations between coregulated genes in the PA14-mapped compendium. (A) Similar to a previously published array compendium, for the PAO1-mapped and PA14-mapped RNA-seq compendia, profile normalization as transcripts per million (TPM), filtering, trimmed-mean-of-means (TMM) normalization, and especially median ratio (MR) normalization correct spurious correlations in the expression of random sets of genes (gray distributions), shifting the medians (green lines) to zero and exposing interoperon gene-gene correlations (black distributions) with elevated medians (purple lines). ***, FDR of <0.01 and large effect size (>0.5) (by a paired Wilcoxon test). (B) For the PA14-mapped compendium, after filtration and MR normalization, samples across the compendium have more similar overall distributions of log_10_ TPM for HK (Housekeeping) genes (purple bars) and 50 randomly selected genes (Random) (green bars). The top annotations of the unfiltered, unnormalized compendium (left heat map) show log_10_ counts of undetected transcripts per profile (Zeros) and the median log_10_ TPM of HK genes colored by whether they were maintained (gray) or removed (orange) in the filtered, normalized compendium (right heat map). (C) Filtering and MR normalization did not alter the results of differential expression analyses examining the effects of low phosphate (Fig. 3C) or manuka honey (Fig. 3E), as seen in the congruence in the log_2_ fold change and −log_10_ FDR values between analyses using compendium-wide normalized data and published count tables. Furthermore, log_2_ fold change values from compendium-derived data MR normalized per experiment (Exp-normed) and across the compendia (Comp-normed) are nearly identical for both data sets examined. Download FIG S2, TIF file, 1.1 MB. Copyright © 2022 Doing et al. 2022 Doing et al. https://creativecommons.org/licenses/by/4.0/ This content is distributed under the terms of the Creative Commons Attribution 4.0 International license.

The high correlations between randomly chosen gene pairs were greatly improved when we applied normalization using the median ratio (MR) method. The MR method is the same as the one employed in the frequently used DESeq2 tool for expression analyses (38) in order to account for differences in read depth (39, 40). Because it produces sample-wise normalization factors using a median-based pseudoreference, it can be easily extended to a compendium of samples with no singular control condition. After normalization of the filtered PAO1-mapped compendium, the correlation between randomly chosen gene pairs was −0.008, while intraoperonic correlations remained high and almost identical to those obtained by the same analysis in the microarray compendium (0.67 for RNA-seq versus 0.66 for microarray) (Fig. 5A). Again, the results were very similar between the PAO1-mapped and PA14-mapped compendia. Interestingly, normalization using another common method, trimmed mean of means (TMM), was less effective (Fig. S2A).

As a further validation of the ability to detect biologically meaningful gene expression correlations across diverse samples, we used 51 manually annotated regulons (each composed of multiple genes, many of which span multiple operons) from the RegPrecise database (v3.2), which contains regulons for many strains, including P. aeruginosa PAO1 (41). These regulons are sets of genes that are regulated by a common transcription factor via conserved promoter motifs. While their expression is not completely linked by shared promoters or by being polycistronic, they are well established to be coregulated, often dynamically in response to environmental cues, and thus present test cases for biological patterns that could be captured by compendia of gene expression. Compared to size-matched sets of random genes, the filtered and normalized PAO1-mapped compendium had high gene expression correlations between genes within a regulon (Fig. 5B). The marked reduction in shared trends in expression between randomly selected genes (green) with each other and trends in expression between randomly selected genes and HK genes (purple) after filtering and normalization can be easily visualized in a heat map of log_10_ TPM values (see Fig. 5C for the PAO1-mapped compendium and Fig. S2B for the PA14-mapped compendium). Additionally, filtering and normalization did not affect the DE analyses of samples from the above-discussed public data sets that examined the transcriptional responses to low phosphate (adjusted *R*^2^ value for log_2_ fold changes of 0.98 [see Fig. 3C for single-experiment analysis]) or manuka honey (adjusted *R*^2^ value for log_2_ fold changes of 0.99 [see Fig. 3E for single-experiment analysis]) (Fig. S2C). The complete filtered and normalized data for all 2,333 profiles derived from mapping to either the strain PAO1 or PA14 cDNA reference genome are referred to as the “PAO1-mapped compendium” and the “PA14-mapped compendium,” and both can be found at the Open Science Framework (OSF) (https://osf.io/vz42h).

Given that both filtration and normalization occur in a compendium-wide manner, we assessed whether the addition of new samples to a compendium changed the overall content. We provide code that allows users to incorporate any data sets from the SRA into a compendium to facilitate the reanalysis of samples of interest whether or not they were captured by the SRA query initially conducted for the purposes of this study or whether samples of interest were excluded based on our filtering criteria. Using this part of the pipeline, we demonstrate that the inclusion of additional samples does not substantially change the filtering threshold values or patterns in gene expression values after compendium-wide normalization. With the addition of 10 new samples, approximately the number of samples in a typical experiment, there was no difference in profiles that were filtered from either compendium. Normalization changed the exact values in the compendia, but individual profiles were significantly correlated, as were principal components calculated from all normalized counts before and after sample additions (Fig. S3).

10.1128/msystems.00341-22.3 FIG S3 The filtering criteria for the compendia of P. aeruginosa gene expression profiles are robust to the addition of new samples. The contents of the PA14- and PAO1-mapped compendia do not substantially change when filtering and normalization criteria are reapplied with 10 new samples added to each compendium. (A) Histograms of *R*^2^ values produced by correlating the normalized counts from a sample in the original compendia with the normalized counts from that same sample in the compendia with 10 additional samples. (B) Correlograms of principal components calculated from the original compendia correlated against principal components calculated from the compendia with 10 additional samples. Both plots show little difference in the final count values. Download FIG S3, TIF file, 0.3 MB. Copyright © 2022 Doing et al. 2022 Doing et al. https://creativecommons.org/licenses/by/4.0/ This content is distributed under the terms of the Creative Commons Attribution 4.0 International license.

### Establishing the potential for cross-compendium analyses using known gene sets.

To further analyze cross-compendium gene expression correlations, 22 gene sets were manually curated from expression profiling analyses of transcription factor mutants, DNA-binding assays, and promoter analyses (promoter fusions and motif searches) performed in a mix of strain backgrounds. The gene sets ranged in size from 5 to 405 genes and spanned multiple transcriptional units, and their gene contents were not exclusive of each other (gene sets are available at the OSF [https://osf.io/5cghu]). These gene sets were involved in global biological programs such as quorum sensing, adaptation to stationary phase, metabolism of specific substrates, and responses to nutrient restriction, oxidative stress, oxygen tension, and virulence-related cues. While gene-gene expression correlations across the compendium were expectedly lower for these gene sets than for operons or regulons, filtering and normalization steps still improved the signal visibility by showing a clear elevation of the median within-gene-set correlation (Pearson correlation coefficient of 0.26) compared to size-matched random controls (Pearson correlation coefficient of −0.001). In paired tests, while 20 of 22 gene sets had significantly higher within-gene-set correlations than the random gene set controls (FDR of <0.01 by a paired Wilcoxon test), there was a wide range of effect sizes. Some gene sets with clearly higher-than-random gene-gene correlations included the extracytoplasmic sigma factors VreI (mean Pearson correlation coefficient of 0.31 [95% confidence interval, 0.28 to 0.33]) and SigX (mean Pearson correlation coefficient of 0.30 [95% confidence interval, 0.27 to 0.33]) and the transcription factors GbdR (mean Pearson correlation coefficient of 0.45 [95% confidence interval, 0.42 to 0.48]) and LexA (mean Pearson correlation coefficient of 0.23 [95% confidence interval, 0.22 to 0.32]) (Fig. 5D). While the distributions of within-gene-set gene-gene correlations varied based on the gene set, some with bimodal distributions (VreI, SigX, and GbdR) and others with more normal distributions (PhoB, AlgU, and LexA), all were remarkably similar across the microarray and RNA-seq compendia (Fig. 5D). Since the microarray and RNA-seq compendia were composed of different samples, the expression relationships between genes that are reflected in both compendia suggest patterns driven by a common influence, which cannot be the expression profiling technology or platform but rather is likely to be the underlying biology. The effectiveness of filtering and normalization to expose compendium-wide gene-gene correlations driven by known biological mechanisms (operons, regulons, and gene sets) foreshadows the potential to identify new biology based on compendium-wide correlations and provides a foundation on which future studies can rely. Genome-wide gene-gene correlation analyses have previously helped uncover new P. aeruginosa biology (37, 42–45) but have yet to be conducted on the scale of the RNA-seq compendia presented here.

Some of the assembled gene sets were large (e.g., PhoB and AlgU, each of which contained hundreds of genes), and while their gene-gene correlations were statistically different than random, the effect size was small, and thus, they did not discriminate well from random control sets. We expect that some of the hundreds of genes in these gene sets include many directly and indirectly controlled genes and genes that are affected in a strain- or condition-specific manner. Such patterns would not be well delineated by these compendium-wide analyses and present an opportunity for future studies to expand upon or further filter these compendia in order to capture specific gene sets with more fidelity. If done so critically, future analyses could also identify the conditions, treatments, or strains necessary to capture such intricate patterns and shed light on biological nuances in the process.

### Analysis of strains, media, treatments, and genetic manipulations profiled in the compendia.

**FIG 6:**
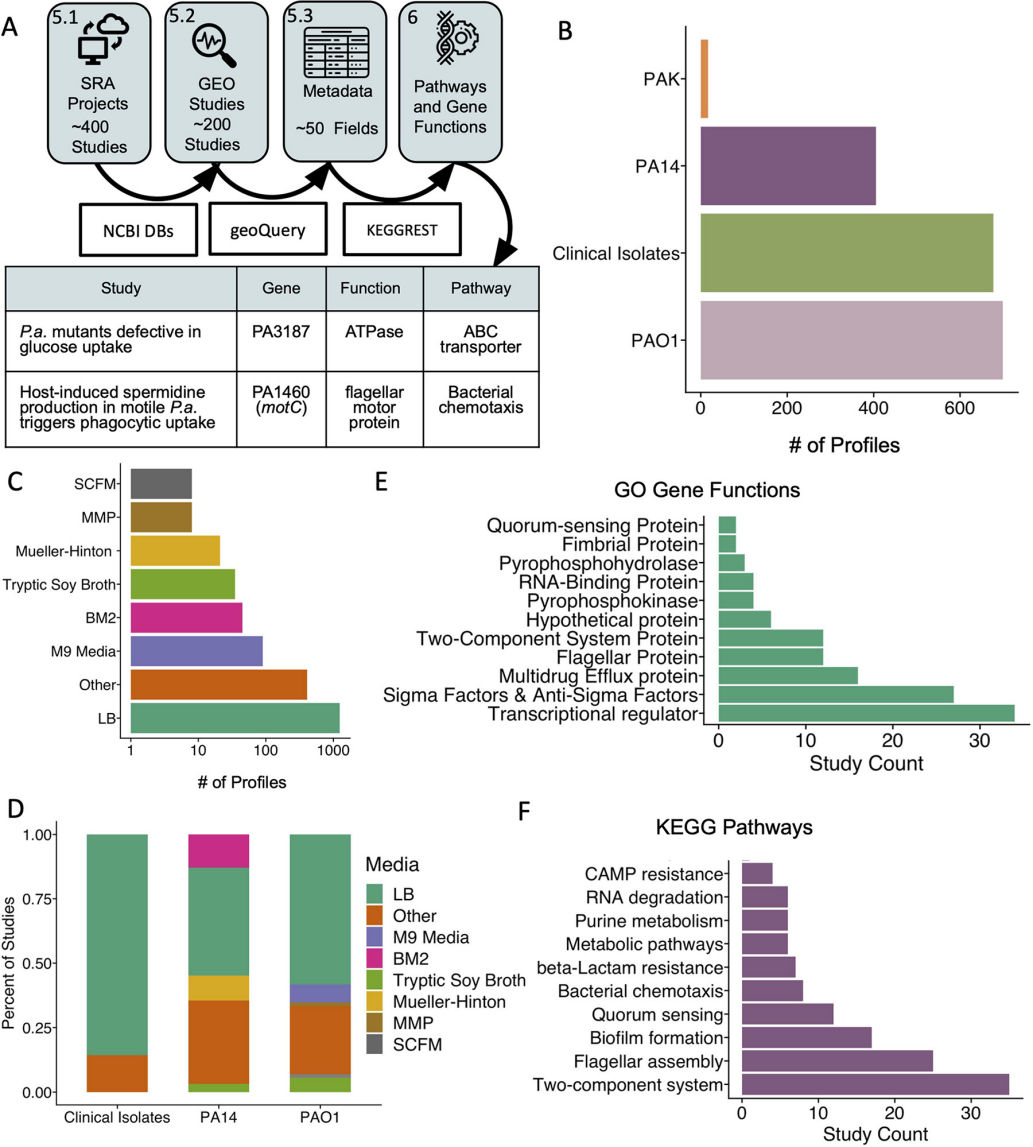
Researchers have interrogated a variety of different P. aeruginosa strains in a range of different media, and a variety of genes have been knocked out or overexpressed. (A) Metadata for SRA studies (step 5.1) also contained in the GEO (step 5.2) were cleaned and curated (step 5.3) prior to the extension of gene annotations to pathways and functions (step 6). DBs, databases. (B) As determined by annotations, PAO1 is the most commonly studied strain, employed in over 70 studies in a total of 646 profiles. (C) LB (lysogeny broth, Lennox broth, and Luria-Bertani) medium is the most common medium used for samples in the compendia. (D) The two most common P. aeruginosa laboratory strains have been studied in various media, and clinical isolates have been predominantly studied in LB medium. (E) Of the biological functions frequently under investigation, transcriptional regulators were the most commonly manipulated GO biological functions, followed by sigma factor or anti-sigma factor genes and multidrug efflux pump genes. (F) Manipulated genes belong to various KEGG pathways, including multiple genes and studies investigating two-component systems. Genes can contribute to multiple pathways.

Curation of metadata is a valuable step in compendium creation because it enables users to visualize trends associated with treatment conditions or strains across multiple studies. To take full advantage of our previously published microarray compendium of P. aeruginosa gene expression data, metadata were manually collected and curated by experts (37). However, because of the time-consuming nature of manual metadata curation, it is a process that is difficult to scale to larger compendia such as the RNA-seq compendia presented here. To meet the challenge of providing curated metadata for the RNA-seq compendia, we employed an R package called GEOquery that automates the collection of metadata associated with studies present in the GEO. Of the 277 BioProject data sets contained in the compendia, about one-half (139) are present in the GEO and therefore have documented metadata amenable to automated parsing (see the workflow in Fig. 6A and the output in Data Set S3). Using these metadata, we analyzed their composition with respect to P. aeruginosa strains. The PAO1-mapped and PA14-mapped compendia, containing the same samples, both encompass approximately 70 studies that used strain PAO1 (646 total profiles) and 30 studies that used strain PA14 (441 total profiles), while the remaining studies used other laboratory strains (PAK or PA3) or clinical and environmental P. aeruginosa isolates (630 profiles) (Fig. 6B).

10.1128/msystems.00341-22.6 DATA SET S3 Full annotations by study. Annotations from studies in the GEO that make up ~50% of the total compendia are shown. Annotations were collected for studies in the GEO including all compiled fields. Download Data Set S3, XLSX file, 0.1 MB. Copyright © 2022 Doing et al. 2022 Doing et al. https://creativecommons.org/licenses/by/4.0/ This content is distributed under the terms of the Creative Commons Attribution 4.0 International license.

We gained further insight into biases in experimental design that might influence gene expression patterns identified in the compendia using the metadata gathered from the GEO that were cleaned and curated. These metadata revealed that studies in the compendia also employed a wide variety of different media. Approximately 70 studies, and nearly 1,250 total profiles, were conducted with LB medium (which includes annotations of lysogeny broth, Lennox broth, and Luria-Bertani medium), which was the most common medium category (Fig. 6C). Other medium types used include minimal media such as M9 minimal medium, BM2 and MMP minimal media for swarming and complex media such as synthetic cystic fibrosis sputum media (SCFM and SCFM2) (46, 47). Approximately one-half of the PAO1 studies and one-half of the PA14 studies used LB medium. Clinical isolates were studied predominantly in LB medium. Some media were used for the study of only one strain. For example, M9 medium was used only in PAO1 studies, while Mueller-Hinton broth was exclusive to PA14 studies (Fig. 6D). Thus, analyses of medium effects for some media should include consideration of the contributions of differences in strain backgrounds represented in different sample sets. A more detailed curation of medium conditions across individual experiments will expand the potential to develop hypotheses based on expression trends.

In several studies, researchers had performed some form of genetic perturbation, the knockout or overexpression of a gene or genes, and then interrogated the effects of this manipulation on P. aeruginosa. Among these studies, there was a notable emphasis on regulators (e.g., transcription factors, sigma factors, and two-component systems) among the associated Gene Ontology (GO) terms (Fig. 6E) and Kyoto Encyclopedia of Genes and Genomes (KEGG) pathways (Fig. 6F). Other functional categories include appendages, biofilm, quorum sensing, drug resistance, and metabolic pathways. An important caveat to keep in mind is that annotations may be incomplete or may reflect indirect or condition-specific effects (Data Set S4). The data and metadata produced by this compendium generation workflow present opportunities for meta-analysis and hypothesis generation to further explore pathways of interest to the community or identify less-well-studied areas, both of which are approaches that could lead down exciting new avenues.

10.1128/msystems.00341-22.7 DATA SET S4 Full functional annotations. KEGG pathway and GO molecular function annotations for studies with aberrant gene expression are shown. Samples are sorted by study or origin; alternately shaded and unshaded rows indicate whether samples were from a common study or not. Download Data Set S4, XLSX file, 0.1 MB. Copyright © 2022 Doing et al. 2022 Doing et al. https://creativecommons.org/licenses/by/4.0/ This content is distributed under the terms of the Creative Commons Attribution 4.0 International license.

## DISCUSSION

Here, we present the largest collection of publicly available P. aeruginosa gene expression data, with twice as many samples as in our previously published microarray compendium (37). Furthermore, this collection is an order of magnitude larger than any other collection of P. aeruginosa RNA-seq data made available to the community (48). Several lessons were learned during the generation of the RNA-seq PAO1-mapped and PA14-mapped gene expression compendia. First, we found that Salmon mapping performs best on microbial gene expression when adjusting parameters to use the unpaired mode, regardless of the paired nature of the data, and decreasing the minimal accepted match length to 15 to allow genomic variation between the reference genome and the samples being processed (49). Second, filtering criteria can be applied to a collection of automatically collected and processed samples to semiautomate quality control processes. Our analyses indicated that too few or too many undetected genes and unexpectedly high or low median values of HK genes were good parameters for filtering out RNA-seq data generated in experiments that did not analyze the whole transcriptome (i.e., chromatin immunoprecipitation sequencing [ChIP-seq] studies) but that application-aware judgments can be made in determining more or less stringent filtering criteria. We found that known gene expression correlations provided excellent metrics for the assessment of filtering and normalization criteria. Third, we found that we could use a common reference genome to analyze transcriptomes from divergent strains. Even within the common laboratory strain PAO1, different samples often contain numerous single nucleotide polymorphisms (SNPs) and genomic variation (50), and P. aeruginosa genomes can be plastic and dynamic (51); thus, it is important to consider reference genome choice, alignment algorithms, and associated parameters. With computationally efficient methods, it is possible to use pangenomes, which capture wide ranges of genetic material from multiple strains (27). In the companion article to this one, Lee et al. explore differences in transcriptional patterns between core and accessory genes of strains PAO1 and PA14, taking an important step in integrating strain-aware analyses with transcriptional profiling (18).

While there will be instances where poor alignment to a reference genome will produce artifacts that could be misleading; the ability to use the Salmon pseudoaligner to rapidly compare the results of mapping single samples to multiple references can reveal when these types of signals arise. The mapping parameters used here are unaffected by the small number of SNPs that distinguish strains. Some SNPs, however, particularly those in global regulators, have large effects on the transcriptome. Because these large transcriptional changes occur via the modulation of other transcriptional regulators that can still activate their cognate regulons, we do not expect that spontaneous functional mutations will negatively impact the ability to detect gene expression relationships in these compendia.

The analysis of these data, which include diverse experiments, strains, medium conditions, and mutants, can greatly aid in the generation of hypotheses that can be rigorously tested by more targeted analyses. We list some potential uses of these compendia below.

(i) As we have reported previously using a compendium of P. aeruginosa microarray data (37, 42–45, 52), compendium-wide analyses can describe regulons that are robust across strains and conditions. These types of analyses can help identify genes that will be most useful as indicators of pathway activities across strains and conditions and in clinical and environmental samples.
(ii) The compendia can be subdivided based on the activity of specific pathways in order to look for other cellular responses that occur when pathways are activated and for the exploration of factors (e.g., strain, medium, or transcriptional activity of other known pathways) that predispose cells to the activation of that pathway.
(iii) If an unknown gene is identified in a phenotypic screen, one can identify conditions under which the gene is most highly expressed relative to all 2,333 other samples. This information can provide insight into function and may indicate experimental systems in which the pathway can be the most easily studied. Furthermore, simple analyses can find genes that most strongly correlate with an unknown gene of interest. The PAO1- and PA14-mapped compendia allow this type of analysis for both core genes and PAO1 and PA14 accessory genes.
(iv) Transcriptomic compendia can be used to gain insights into genotypes. Strains with frequent naturally occurring mutations in genes encoding transcriptional activators can have characteristic signatures. For example, *lasR* loss-of-function mutants will have low levels of target genes such as *lasI* and *lasB*, strains with mutations in genes encoding repressors like *mucA* often lead to high *alg* gene expression levels, and mutations in genes encoding regulators such as *mexZ* have higher expression levels of genes encoding drug efflux pumps like *mexXY*. Thus, researchers may find these compendia useful for analyzing a clinical isolate in the context of all other strains to predict genotypes. Furthermore, since these compendia are comprised exclusively of publicly available data, the opportunity to download the reads to look for individual SNPs exists.

The workflow presented here can be redeployed to create other compendia. These compendia were constructed by mapping to cDNA reference genomes. To gain better insight into the expression of identical or nearly identical paralogs, a reference genome that includes 5′ untranslated regions may provide insight into the differential expression of these loci. Other reference genomes, such as one with accessory genes from many different P. aeruginosa strains, could be used. This approach will be particularly useful as the number of different strains analyzed by RNA-seq increases. There are now multiple microbes for which there are thousands of publicly available transcriptome data sets, including Escherichia coli, Staphylococcus aureus, Saccharomyces cerevisiae, and Candida albicans. The workflow presented here is scalable and adaptable to new data sets and organisms and provides a critical approach to fully utilizing public transcriptomics data.

## MATERIALS AND METHODS

### RNA-seq sample collection and processing.

The RNA-seq data set used to compare CLC and Salmon alignments consisted of wild-type PA14 and the *pstB*::Tn*M* mutant grown as colony biofilms on plates containing 3-(*N*-morpholino)propanesulfonic acid (MOPS), 0.2% glucose, and 0.7 mM phosphate with 1.5% agar for 16 h. Duplicate samples were obtained for each strain. Cells were collected as cores from agar plates: cores were taken using a straw, and cells were suspended by shaking agar plugs in 1 mL of distilled water (dH_2_O) on a Disrupter Genie instrument for 3 min. RNA was isolated using the Qiagen RNeasy kit (catalog number 74004) and DNase treated using the Turbo kit (catalog number AM2239). Libraries were prepared according to Illumina protocols, including ribodepletion, and sequenced on the Illumina NextSeq platform at the Geisel School of Medicine Genomics Shared Resource. RNA-seq data were processed using CLC Genomics Workbench v12 using default settings, as described previously (45), with reference genomes for PAO1 (NCBI reference sequence accession number NC_002516.2) and PA14 (NCBI reference sequence accession number NC_008463.1), and differential expression analysis was performed using EdgeR (26).

### Validation of Salmon mapping methodology by analysis of public data.

For analyses of publicly available data, reads and the corresponding data tables for counts were downloaded from the SRA and the Gene Expression Omnibus (GEO), respectively. For the samples shown in Fig. 3C and D, samples with SRA accession numbers SRX474130 and SRX474131 (control conditions) and SRX474128 and SRX474129 (low phosphate) were mapped to PA14 using Salmon as part of the compendium construction pipeline. In the published data, reads had been aligned to genome sequences for strain PA14 using stampy (53). For the analyses shown in Fig. 3E and F, samples with SRA accession numbers SRX7423386, SRX7423388, and SRX7423390 (control conditions) and SRX7423383, SRX7423384, and SRX7423385 (with manuka honey) were used and were also mapped to PA14 using the compendium pipeline, and the published reads (32) had been aligned to the P. aeruginosa UCBPP-PA14 genome sequence using RSubread (v1.30.7). For the samples shown in Fig. 3G and H, samples with SRA accession numbers SRX1017135 and SRX1017136 (clinical isolate J215) and SRX1017137 and SRX1017138 (J215 Δ*anr*) were mapped to PAO1 using Salmon as part of the compendium pipeline, and published read counts were aligned using CLC Genomics Workbench as described previously (33).

### Salmon mapping to create PAO1-mapped and PA14-mapped compendia.

Salmon (v1.5.2) was run in mapping-based mode to make use of its fast-mapping algorithm. To create the transcriptome indices using cDNA, references were obtained from the Ensembl bacterial database (release 54) FTP site (PAO1 cDNA sequences were sourced from assembly ASM676v1 with NCBI genome accession number GCA_000006765 as annotated for coding regions under BioProject accession number PRJNA331 to include cDNA sequences under GenBank accession numbers AAG03391 to AAG08955, and PA14 cDNA sequences were sourced from assembly ASM1462v1 of with NCBI genome accession number GCA_000014625 as annotated for coding regions under BioProject accession number PRJNA386 to include cDNA sequences under GenBank accession numbers ABJ09812 to ABJ15703), as maintained by PseudoCAP and sourced from the Pseudomonas Genome Database. The Salmon index call was used with 15 set as the minimum length for an acceptable alignment match (k = 15). To map reads, the Salmon quant call was used, with the validation method set to “score” and “validate mappings.” Code, including links to necessary reference data files, is available at https://github.com/hoganlab-dartmouth/pa-seq-compendia, and cDNA reference files as well as all generated data are available at https://osf.io/s9gyu/ in an Open Science Framework project. After the filtering heuristic step, there were 2,333 samples separately mapped to 5,563 genes using the PAO1 cDNA reference genome and 5,891 genes using the PA14 cDNA reference genome to create the PAO1-mapped and PA14-mapped compendia, respectively.

### Determination of heuristic criteria for filtering samples from the compendia.

Housekeeping genes (*ppiD*, *rpoD*, *proC*, *recA*, *rpsL*, *rho*, *oprL*, *tpiA*, and *nadB*) were chosen from the literature and P. aeruginosa gene expression field standards (54, 55). Percentile cutoffs were based on visual inspection and the removal of data sets with technical differences (metatranscriptomic data and RIP-seq data). The sparsity values reflect the number of genes with “zero” counts. The samples (both those retained and those removed) from the compendia based on these values are indicated in Data Set S2 in the supplemental material. The values for samples removed based on the filtering criteria are indicated in Fig. 5C.

### Compendium normalization.

Transcripts per million (TPM) and transcript counts (counts) are estimated by Salmon and exported directly. Trimmed-mean-of-means (TMM) and ratio-of-medians (RM) normalization methods were applied to the estimated counts exported from Salmon using the R packages EdgeR (26) and DESeq2 (38), respectively. For both normalization methods, per-sample coefficients were extracted and multiplied by the estimated counts. After normalization, the compendia were scaled from 0 to 1 with a linear transformation based on the matrix maxima and minima. The array compendium was downloaded from the ADAGE GitHub repository (https://github.com/greenelab/adage) and had already been scaled from 0 to 1 with linear transformations based on gene-wise maxima and minima. The filanl-filtered and MR-normalized compendia are provided at the OSF (https://osf.io/bj9mx [mapped to strain PAO1] and https://osf.io/vnd68 [mapped to strain PA14]).

### Comparison of filtration and normalization steps by correlation analyses.

Pearson correlation coefficients were calculated in R using the base cor function on predetermined sets of genes (computationally predicted operons from the Pseudomonas Genome Database [10], regulons from RegPrecise [41], and gene sets from select publications [see the OSF project at https://osf.io/7jrg8/ for operons, regulons, and gene sets with references]) and size-matched sets of randomly selected genes from the row names of the compendia. Distributions and medians were plotted in ggplot2 (56). Operons from the Pseudomonas Genome Database include those in DOOR (Database for Prokaryotic Operons), which uses sequence features, including intergenic distance, conservation of neighboring genes across genomes, and phylogenetic distance, to classify genes as being cooperonic, and PseudoCAP, which provides annotations based on manually reviewed literature (57, 58).

The effect of adding new samples was assessed after the addition of 10 new samples. Exact cutoff values were recalculated using the same percentiles of zero counts (10th and 90th) and housekeeping gene expression (20th and 98th) and applied to the compendium with the additional 10 samples. Filtration was reapplied to the entire compendia, performed on the raw NumReads estimates produced by Salmon, and the numbers of samples that were retained in the compendia were compared. Normalization was performed on the filtered data sets. For each sample, the resulting normalized read counts were correlated between the original and new compendia using the R function cor. A principal-component analysis was performed on each compendium using the R function prcomp. The resulting principal components were compared between the original and new compendia using the R function cor.

### Annotations.

Detailed metadata were gathered for all of the P. aeruginosa RNA-seq studies present in the GEO with select fields, including strain, media, genetic perturbation, and other experimental conditions. P. aeruginosa RNA-seq studies were identified in the GEO by searching for “Pseudomonas aeruginosa” in the GEO Data Set (GDS) browser, filtering by the study type “expression profiling by high-throughput sequencing,” and further filtering by organism to select only studies containing P. aeruginosa RNA-seq data (i.e., not RNA-seq studies of mice or human cells exposed to P. aeruginosa, which came up with the basic search).

Next, a summary file containing very limited metadata for each P. aeruginosa RNA-seq study was downloaded directly from the GEO as a .txt file. This summary contained an FTP download link for each study, in which the GEO series accession number for the study was embedded. For all studies, the accession number was extracted with the str_extract_all function of the R package stringr (59) and subsequently fed into the getGEO function of the GEOquery R package from Bioconductor (60). The getGEO output is a large R list object containing very detailed metadata on the strains, media, treatments, and other conditions employed for each study.

With detailed metadata in hand, certain fields of interest were extracted, organized into columns in an R data frame, and exported from R as a .csv file. This .csv file was further cleaned in Excel so that the metadata could be reuploaded to R in a format suitable for figure creation. All figures involving annotation data were created with the R package ggplot2 (56). Additional information was gathered from the Kyoto Encyclopedia of Genes and Genomes (KEGG) Rest server using the KEGGREST package from Bioconductor (61). For P. aeruginosa RNA-seq studies that perturbed certain genes, the common gene names provided in the metadata (e.g., *sigX*) were converted manually to their respective locus tags (e.g., PA1776) and fed into KEGGREST’s keggGet function, which extracted information on associated KEGG pathways and gene functions.

### Data availability.

All processed data, including the final, filtered, normalized PAO1-mapped and PA14-mapped compendia as well as the unfiltered, unnormalized compendia and intermediate files, are available as an Open Science Foundation project (https://osf.io/s9gyu/), and code necessary to recapitulate analyses are available at the pa-seq-compendia GitHub repository (https://github.com/hoganlab-dartmouth/pa-seq-compendia). Raw data for the original RNA-seq analyses presented in this study are available in the NCBI SRA database and the Gene Expression Omnibus under accession number GSE192694.
