# Supplementary figures and images for: Computationally Efficient Assembly of Pseudomonas aeruginosa Gene Expression Compendia
Source: mSystems. 2022 Dec 21;8(1):e00341-22. doi: 10.1128/msystems.00341-22 (PMC9948711; doi:10.1128/msystems.00341-22)

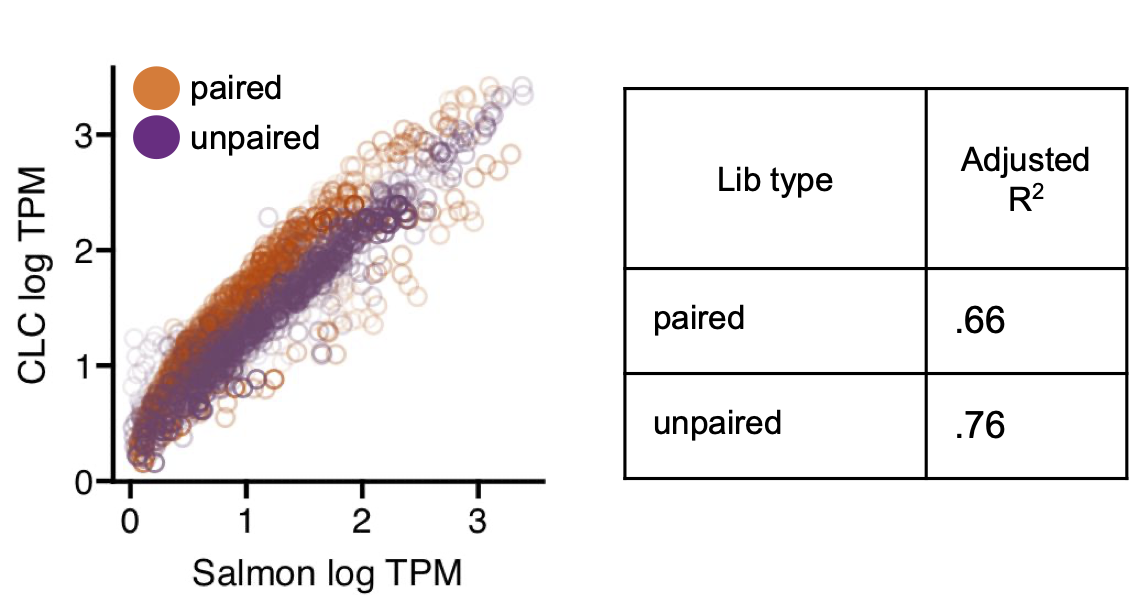

Supplement: FIG S1 [file msystems.00341-22-s0001.tif]
